# Acute kidney injury management using intermittent low efficiency haemodiafiltration in a critical care unit: 39 dogs (2012–2015)

**DOI:** 10.1186/s13028-019-0452-6

**Published:** 2019-04-10

**Authors:** Maxime Cambournac, Isabelle Goy-Thollot, Julien Guillaumin, Jean-Yves Ayoub, Céline Pouzot-Nevoret, Anthony Barthélemy, Jeanne-Marie Bonnet-Garin

**Affiliations:** 1 Intensive Care Unit (SIAMU), Université de Lyon, VetAgro Sup, APCSe, 69280 Marcy l’Etoile, France; 2 Emergency and Critical Care Service, Department of Veterinary Clinical Sciences, The Ohio State University, Columbus, OH 43210 USA; 3 Physiology Unit, Université de Lyon, VetAgro Sup, APCSe, 69280 Marcy l’Etoile, France

**Keywords:** Acute renal failure, Outcome, Prognostic factors, Renal replacement therapy, Urinary output

## Abstract

**Background:**

Veterinary studies describing acute kidney injury (AKI) management using renal replacement therapy (RRT) are limited and have primarily focused on intermittent haemodialysis in North American populations. European data are lacking, although differences in populations, pathogen and toxin exposure and RRT modalities may exist between Europe and North America. The present study reviewed RRT-managed cases from the intensive care unit (ICU) of VetAgro Sup, Lyon, France, for the period 2012–2015. The aims were to describe a 4-h RRT protocol of intermittent low efficiency haemodiafiltration, population characteristics and outcomes in canine AKI cases requiring RRT and to identify prognostic variables. We defined DeltaCreat/h as the difference between the serum creatinine level after RRT treatment N and that before treatment N + 1 divided by the time between treatments (in hours).

**Results:**

Thirty-nine dogs were included, and 67% were males. The median (range) age, weight, hospitalization length and number of RRT treatments were 4.4 (0.25–15) years, 26.6 (6.7–69) kg, 8 (1–23) days and 3 (1–8) treatments, respectively. The main AKI causes were leptospirosis (74.4%) and nephrotoxins (15.4%). Age (4.0 vs 5.4 years; P = 0.04), admission urine output (0.5 mL/kg/h vs 0 mL/kg/h; P = 0.02) and hospitalization length (10 vs 4 days; P < 0.001) differed between survivors and non-survivors. Hospitalization length [odds ratio (OR) = 0.4], number of treatments (OR = 5.1), serum potassium level on day 2 (OR = 1.9), DeltaCreat/h between the first and second treatments (OR = 1.2), and UOP during hospitalization (OR = 0.2) were correlated with outcome. The main causes of death were euthanasia (44%) and haemorrhagic diatheses (33%). The overall survival rate was 54%, with 55% of survivors discharged with a median creatinine < 240 µmol/L.

**Conclusions:**

This is the first description in the veterinary literature of a 4-h protocol of intermittent low efficiency haemodiafiltration to provide RRT in a veterinary critical care unit. While this protocol appears promising, the clinical application of this protocol requires further investigation. Among parameters associated with survival, UOP and DeltaCreat/h between the first and second RRT treatments may be prognostic indicators. The applicability of these parameters to other populations is unknown, and further international, multicentre prospective studies are warranted to confirm these preliminary observations.

## Background

Diagnosing and managing critically ill patients with renal dysfunction alone or as part of multiple organ dysfunction syndrome is a part of the daily routine in intensive care units (ICUs) [1]. Mortality due to acute kidney injury (AKI) has been reported to be ranging from 23.8 to 78.5% in dogs [2–4]. Despite adequate medical management, renal replacement therapy (RRT) may be necessary to support the consequences of severe AKI [3]. Considered standard in human hospitals, few veterinary facilities are equipped to provide RRT in Europe. As some such facilities are trying to establish an RRT program, various factors must be considered, such as availability, expertise, facility settings, resources, and costs [5]. Intermittent haemodialysis (IHD) requires a sizeable investment to purchase and maintain specialized water treatment facilities, whereas continuous renal replacement therapy (CRRT) uses pre-packaged sterile fluids [6, 7]. Because newest CRRT machines allow to perform haemoperfusion and therapeutic plasma exchange on the same platform, they are now preferred in 80% of human ICUs [7]. However, CRRT is technically demanding, often associated with the need for continuous anticoagulation, and requires 24-h supervision, which is costly [8]. Although similar to other modalities regarding patient outcomes, intermittent RRT protocols are becoming increasingly popular in critically ill humans with AKI because it reduces care complexity compared to CRRT [9]. Although costs and staff are important concerns in veterinary medicine, a 4-h intermittent low efficiency haemodiafiltration treatment may be a valuable option in veterinary ICU settings with limited staffing. To the authors’ knowledge, no such protocol has yet been published so far in the veterinary literature. While veterinary guidelines on the dose and the timing of initiation can be found elsewhere [5], only few studies have described the use of RRT for managing veterinary AKI patients, and all are focused on North American patient populations [10–15]. If and when a pet will recover kidney function are frequent questions in veterinary medicine, but causes of AKI, procedures specificity and survival rate may vary among centres. The present study describes a new specific 4-h intermittent low efficiency haemodiafiltration protocol to provide RRT in a veterinary ICU, and report characteristics, clinical features, aetiologies and outcomes in a European canine population with RRT-managed AKI.

## Methods

### Case selection

Medical records of dogs presented at the ICU (SIAMU) of VetAgro Sup, Campus vétérinaire de Lyon, France, between January 2012 and January 2015 were retrospectively reviewed. All dogs included in the study were required to be admitted to our ICU for AKI and to have received at least one RRT treatment. AKI was defined as an abrupt reduction in kidney function recognized by an increased serum creatinine concentration or reduced urine output (UO) [16]. Grading and classification of AKI severity was based on the International Renal Interest Society^1^ (IRIS) AKI grading scheme. Exclusion criteria were missing data (up to 2 values for 2 parameters) and suspicion or diagnosis of chronic kidney disease (CKD) based on historical findings (polyuria, polydipsia, weight loss, and documented previous azotaemia) or ultrasonographic changes consistent with chronic parenchymal lesions.

### Acute kidney injury causes

Dogs were diagnosed with leptospirosis if they fulfilled at least 1 of the following 3 criteria: single microscopic agglutination test titres > 1:800 for nonvaccine serovars or > 1:1600 for vaccine serovars; a fourfold rise in convalescent titres; and a positive urine or blood polymerase chain reaction assay as described previously [17]. Obstructive urolithiasis was diagnosed based on ultrasound findings. Leishmaniasis was diagnosed based on positive ELISA^2^ results together with negative results for other blood-borne parasites. As dogs with leishmaniosis might be co-infected with other vector borne diseases or suffering from other concomitant infectious or non-infectious diseases making, standard procedure in our unit is to rule out any co-infections or other blood-borne parasites [18]. Intoxication was considered based on history (owner witnessing ingestion or finding toxic product box opened or chewed) and clinical signs consistent with intoxication and after excluding infectious and congenital causes.

### Variable acquisition

General data included signalment; body weight (BW), blood pressure and volume status at admission; time to referral, defined by the time between first signs (described by the owner or the regular veterinarian) and admission to our facility; hospitalization length; time from admission to initiation of RRT; cause of AKI; number of RRT treatments; hospital discharge status (survivors [S] or non-survivors [NS]); and causes of death (natural or euthanasia).

Volume status at admission was classified as normal, hypovolaemia or overfilling.

Hypovolaemia was considered if physical signs consistent with hypovolaemia (pale mucous membrane, capillary refill time > 2 s, decreased peripheral pulse quality) were present.

Overfilling was considered if physical signs consistent with volume overload (acute weight gain, jugular retrograde pulse, peripheral oedema, or increased skin elasticity) were present.

Urine output (UOP) data included admission UOP, defined as the mean urine production during the first 8 h of hospitalization, after aseptic placement of the indwelling urinary catheter^3^ and complete bladder emptying, and daily UOP as the median of the recorded values for each individual day.

Electrolytes, acid–base and venous blood gas data included pH; PCO_2_; bicarbonate; anion gap; and sodium, potassium and chloride concentrations. If more than one analysis was performed per day, only the RRT pre-treatment test results were recorded.

Serum biochemistry data included blood urea nitrogen (BUN) and serum creatinine concentrations at admission, before and after each RRT treatment or daily if no RRT treatment occurred. The urea reduction ratio (URR) was calculated as follows: URR = (1 − BUNpost/BUNpre) * 100, where BUNpre and BUNpost represent the pre- and post-treatment BUN concentrations, respectively. Based on serum creatinine values, creatinine reduction ratio (CRR) was calculated using the same formula. DeltaCreat/h (N, N + 1), defined as the difference between serum creatinine after RRT treatment N and before treatment N + 1 divided by the time (in hours) between the 2 treatments, expressed in µmol/L/h, reflected the inter-treatment kinetics of creatinine concentration variation.

### Case management and RRT indications

All patients were initially treated with intravenous fluids to adequately restore perfusion parameters and resolve dehydration if necessary. All additional treatments were at the discretion of the attending clinician, and included antimicrobial therapy (amoxicillin with clavulanic acid), proton pump inhibitor, antiemetic, anti-diarrheic, and specific symptomatic treatments as needed.

RRT treatment was considered in cases of ingestion ethylene glycol (and derivatives) or grapes, worsening or lack of significant improvement in BUN (> 35 mmol/L) or serum creatinine concentration (> 400 µmol/L), refractory medical hyperkalaemia (> 6.5 mmol/L) or rapidly rising values, uncompensated refractory metabolic acidosis (pH < 7.1), and evidence of fluid overload refractory to diuretics or oligoanuria (UOP < 0.3 mL/kg/h) according to the modern criteria for initiating RRT in the ICU [19].

Refractory hyperkalaemia was defined as a persistent serum potassium > 6.5 mmol/L, despite the following treatments: salbutamol (2 doses of 100 µg/dose with space chamber every 15 min for 1 h, then 2 doses every hour if potassium > 6.5 mmol/L), insulin and glucose (0.5 units/kg regular insulin IV and, for every unit of insulin administered, 2 g/UI of 50% dextrose diluted IV to prevent hypoglycaemia), sodium bicarbonates (1–2 mEq/kg IV slowly over 15 min). If still refractory, a rescue solution containing 400 mL of 25% dextrose, 50 U of regular insulin, 50 mmol of sodium bicarbonate in 1 litre bag of normal saline was used if RRT was not available [20].

Refractory metabolic acidosis was defined as a persistent pH < 7.1 with concurrent bicarbonate value < 12 mmol/L despite administration of 100% of base deficit over 2 h.

### RRT technique

Venous access was provided by temporary two-lumen dialysis catheters^4^ ranging in size from 7 to 11.5-French. The distal tip of the catheter was advanced to the level of the right atrium when possible or the cranial vena cava when the catheter length precluded atrial placement. Thoracic radiography was used to confirm appropriate catheter tip location.

One CRRT machine^5^ was used in CVVHDF mode with commercially available standard balanced electrolyte dialysate solutions.^6^ Preconnected filter kits were adapted to the patient’s BW.^7^ Treatment goals were to reduce BUN and creatinine concentrations by one-third to one-half. Blood flow was usually started at 1 mL/kg/min to decrease dialysis initiation hypotension and assess tolerance, then progressively increased to 8 mL/kg/min [21, 22]. Dialysate flow rate ranged from 1000 to 2500 mL/h. The ultrafiltration (UF) rate setting and total patient removal volume were based on the clinician’s fluid status evaluation of the patient. For each treatment, potassium chloride was added to the same standard dialysate solution if needed, following Brenner’s recommendation, commonly referred to as the “rule of seven”, defined as [serum potassium] + [dialysate potassium] = 7 mmol/L [22]. The anticoagulation protocol was based on circuit priming with 1000 mL of 0.9% NaCl^8^ containing 2500 units/L of unfractionated heparin and a patient initial anticoagulation with an initial bolus of 50 UI/kg of unfractionated heparin.^9^ Intermittent boluses of 10–30 UI/kg were used at mid-treatment. Additional heparin boluses were administered in case of direct clotting visualization in the circuit, dark streaks in the dialyzer, foaming or clot formation in the venous trap, clots at the arterial header or if transmembrane pressure increased above 220 mmHg [23]. Sessions lasted between 4 and 5 h because of medical staff constraints but may have ended earlier in cases of worsening clinical conditions or severe clotting. ICU technicians performed all post-treatment blood analysis 1 h after the end of an RRT treatment according to the in-house standard procedure.

### Statistical methods

For statistical analysis, aetiology was divided between leptospirosis and non-leptospirosis, and outcome between S and NS at discharge, regardless of the cause of death. Descriptive statistics were used for categorical variables. The D’Agostino–Pearson omnibus test was used to assess normality. For ease and consistency, all continuous variables were presented as the median (range). Differences between variables were assessed by comparing the rank distributions with the non-parametric Mann–Whitney *U* test. Considering the number of data and the potential bias associated with unassessed confounding factors in the univariate analysis, only independent predictors from the multivariate analysis are presented [24]. Statistical associations for all parameters with outcome were determined by forward stepwise regression, a method for regressing multiple variables while excluding those that are not statistically significant [25]. The significance level for addition to the regression was fixed for each variable with a Wald score P value < 0.2. For significant categorical data, odds ratios (ORs) and their associated 95% confidence intervals (CIs) were calculated and receiver operating characteristic (ROC) curves were generated. Cut-off values that maximized specificity and sensitivity were determined based on the Youden index. The goodness of fit of the final model was evaluated using the Hosmer and Lemeshow test [26]. Statistical significance was set to 0.05 for all tests. All descriptive statistics were analysed and graphs constructed using commercial software.^10^

## Results

### Population characteristics

Forty-three dogs were eligible for the study. Four dogs were excluded because of incomplete medical records (n = 1), diagnosis of CKD (n = 2) or suspicion of CKD based on small and irregular kidneys, with diffusely poor echogenicity and loss of the corticomedullary junction on abdominal ultrasound (n = 1). Therefore, data on 39 dogs are presented below for a total of 116 RRT treatments.

Demographic data are reported in Table 1. Neuter status was 4 (10%) castrated and 21 (54%) intact for males and 7 (18%) spayed and 7 (18%) intact for females. Breeds included mixed breed (n = 8); Labrador Retriever (n = 3); two each of American Staffordshire Terrier, German Shepherd, Anatolian Shepherd, Border Collie, Golden Retriever, French Bulldog, Bernese Mountain, Cane Corso, and Jack Russell Terrier; and one each of Boxer, Briard, Cairn Terrier, English Cocker Spaniel, American Cocker Spaniel, Shi Tzu, Landseer, Canary Mastiff, French Mastiff, and Brittany Spaniel. NS dogs were significantly older than S dogs (5.4 [range: 0.3–15] years vs 4 [range: 0.3–10] years; P = 0.04; Table 1).

**Table 1:** Description of demographic and laboratory data (median and range) for 39 dogs with acute kidney injury and comparison between survivors and non-survivors

	All (n = 39)	Survivors (n = 21)	Non-survivors (n = 18)	P
Demographics				
Age (years)	4.4 (0.25–15)	4 (0.25–9.8)	5.4 (0.3–15)	0.04*
BW (kg)	26.6 (6.7–69)	30.8 (6.9–69)	24 (6.7–67)	0.30
Male (%)	26 (66%)	15 (71%)	11 (61%)	0.48
Laboratory data at admission				
BUN (mmol/L)	52 (17.5–97.4)	46.4 (17.5–82.1)	56.8 (37.2–97.4)	0.43
Creat (µmol/L)	790 (235–1951)	785 (235–1682)	927 (260–1951)	0.16
ALT (IU/L)	137 (38–1739)	145.5 (38–1705)	127 (42–1739)	0.48
AlkP (IU/L)	84 (26–416)	81 (26–416)	85 (36–416)	0.85
Total protein (g/dL)	54 (33–81)	55 (33–81)	54 (38–69)	0.69
Na^+^ (mmol/L)	152 (135–178)	150 (135–178)	152 (135–176)	0.56
K^+^ (mmol/L)	4.7 (2.7–9.1)	4.4 (2.7–9.1)	4.9 (3.4–7.9)	0.10
Cl^−^ (mmol/L)	112 (98–127)	112 (100–125)	111.5 (98–127)	0.80
pH	7.32 (7.02–7.52)	7.34 (7.02–7.52)	7.29 (7.07–7.41)	0.21
pCO_2_ (mmHg)	34 (22–52)	36 (23–50)	33 (22–52)	0.71
HCO_3_^−^ (mmol/L)	17.15 (7.8–27.5)	18.1 (10.3–27.5)	17.1 (7.8–23)	0.35
Anion gap	27.4 (17–49.3)	25.4 (17–34.8)	29 (21.9–49.3)	0.01*

*BW* body weight, *BUN* blood urea nitrogen, *Creat* creatinine, *ALT* alanine amino transferase, *AlkP* alkaline phosphatase

*Significant difference between survivors and non-survivors

Clinical signs at admission included lethargy (92.3%, 36/39), anorexia (87.1%, 34/39), and vomiting (74.3%, 29/39). The median rectal temperature was 37.8 (range: 36.8–39.8) °C. The median heart rate was 90 (range: 60–200) beats/min. The median respiratory rate was 30 (range: 20–60) breaths/min. Dyspnoea was noted in 6 dogs (15.3%), with inspiratory dyspnoea in 3/6 (50%) and expiratory dyspnoea in 3/6 (50%). The median blood pressure was 145 (range: 110–195) mmHg.

Regarding volume status at admission, 19 (48.7%) dogs presented physical signs consistent with volume overload, and none with hypovolaemia. The remaining dogs (51.3%, 20/39) were classified as normovolemic. There was no significant difference of any of the clinical signs between survivors and non-survivors.

### Acute kidney injury causes

Causes of AKI requiring RRT were leptospirosis (74.4%, 29/39), intoxication (15.4%, 6/39; grape [4/6], ethylene glycol [1/6] and gentamicin [1/6]), unknown aetiology (5.1%, 2/39), leishmaniasis and bilateral obstructive ureterolithiasis (2.6%, 1/39 for each; Table 2). The dog with bilateral ureterolithiasis received RRT as a bridge to bilateral subcutaneous ureteral bypass surgery. The dog with ethylene glycol intoxication received RRT based on short time between ingestion and presentation, as well as owner demand to treat.

**Table 2:** Medical data (median and range) for 39 dogs with acute kidney injury and comparison between survivors and non-survivors

	All (n = 39)	Survivors (n = 21)	Non-survivors (n = 18)	P
Medical data				
Aetiology				
Leptospirosis	29 (74%)	16 (76%)	13 (72%)	
Intoxication	6 (15%)	3 (14%)	3 (18%)	
Other	4 (10%)	2 (10%)	2 (11%)	
Time to referral (days)	4 (0–31)	5 (2–31)	3 (0–12)	0.1
Hosp. length (days)	8 (1–23)	10 (4–23)	4 (1–14)	< 0.001*
RRT data				
Time to initiation (days)	18 (4–72)	18 (6–48)	18 (4–72)	0.7
Number of RRT treatments	3 (1–8)	2 (1–8)	3 (1–7)	0.32
DeltaCreat/h (1, 2)	5.7 (− 13.5 to 71.8)	0.8 (− 13.5 to 29.9)	10.7 (− 10.7 to 71.8)	0.002*
DeltaCreat/h (2, 3)	14 (− 100 to 117)	2 (− 100 to 82)	28 (− 49 to 117)	0.04*
DeltaCreat/h (3, 4)	− 2 (− 104; 100)	− 8 (− 61 to 5)	4 (− 104; 100)	0.05*
UOP at adm.				
Anuric	13 (35.1%)	4 (10.8%)	9 (24.3%)	0.015*
Non-anuric	24 (64.9%)	18 (48.6%)	6 (16.2%)	0.015*
UOP (mL/kg/h) in hosp.				
Day 1 (n = 37)	0.5 (0–10)	0.5 (0–10)	0 (0–2)	0.02*
Day 2 (n = 37)	1.8 (0–16.3)	2.4 (0–16.3)	0.2 (0–3)	< 0.0001*
Day 3 (n = 36)	1.95 (0–14.2)	3 (0–12)	0.3 (0–2.29)	< 0.0001*
Day 4 (n = 30)	2.8 (0–12)	3 (7–12)	0.4 (0–4)	< 0.0001*
Day 5 (n = 28)	3.2 (0–7.5)	3.1 (1–7.5)	0.4 (0–4)	0.0003*

RRT: extracorporeal renal replacement therapy. UOP at adm.: urine output at admission, with anuria defined as UOP < 0.3 mL/kg/h. DeltaCreat/h (1–2), (2, 3) and (3, 4): difference between serum creatinine concentration in µmol/L/h after the first and before the second, after the second and before the third, and after the third and before the fourth RRT treatment, respectively, divided by the time (in hours) between treatments. UOP in hosp.: daily urine output during hospitalization. Hosp. length: hospitalization length, in days

*Significant difference between survivors and non-survivors

### Hospitalization data

Median hospitalization length was shorter for NS than for S [4 (range: 1–14) days vs 10 (range: 4–23) days; P < 0.001; OR = 0.4; 95% CI 0.2–0.7; Tables 2 and 3]. In the multivariate analysis, the number of RRT treatments was significantly correlated with a negative outcome, with NS receiving more treatments than S (OR = 5.1; 95% CI 1.7–16.0; Table 3).

**Table 3:** Summary of significant prognostic factors’ characteristics identified in the multivariate analysis

Parameter	Odds ratio	95% Wald confidence interval		
Number of RRT treat.	5.1	1.65	16	
Hospitalization length	0.4	0.2	0.7	

Parameter	Odds ratio	Sensitivity (%)	Specificity (%)	AUC	Cut-off
UOP (day 3)	0.2	76.9	90	0.9	1 mL/kg/h
K (day 2)	1.9	53	90	0.7	5.3 mmol/L
DeltaCreat/h (1–2)	1.2	92	63	0.8	1.83 µmol/L/h

Number of RRT treat.: number of extracorporeal renal replacement therapy (RRT) treatments. UOP (day 3): median urinary output at day 3. K (day 2): serum potassium concentration at day 2. DeltaCreat/h (1–2): difference between serum creatinine concentration in µmol/L/h after the first and before the second RRT treatment divided by the time (in hours) between the 2 treatments

### Urine output

UOP at admission was not available for 2 dogs. The median UOP at admission was significantly higher in the S group than in the NS group (0.5 mL/kg/h (range: 0–10) vs 0 mL/kg/h (range: 0–2); P = 0.02; Table 2). Nine dogs in the NS group were anuric, compared to 4 dogs in the S group (P = 0.015). Anuria at admission was significantly associated with a negative outcome (OR = 6.8; 95% CI 1.4–24.3).

**Fig. 1:**
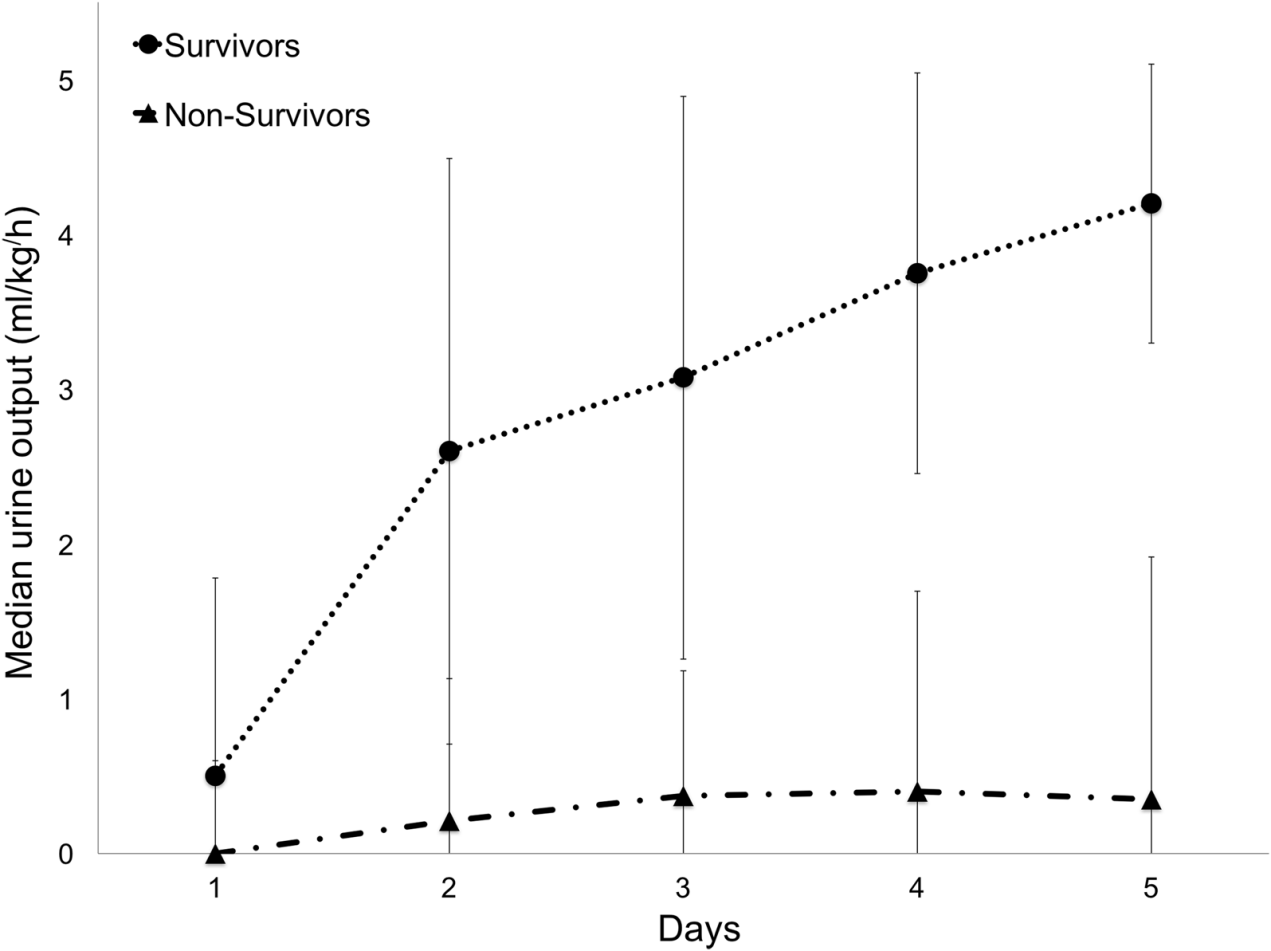
Daily urine output (median ± range) for survivors (dotted line, circle mark) and non-survivors (broken line, triangle mark). *Significant difference; ^✝^predictive capacity

From day 1 to day 5, UOP was significantly higher in the S group than in the NS group (P ≤ 0.02 for each individual day) (Table 2, Fig. 1). In the multivariate analysis, elevated UOP at day 3 was associated with a positive outcome (OR = 4.5; 95% CI 1.5–14.0). Based on the ROC curve analysis, the optimal UOP at day 3 cut-off value of 1 mL/kg/h maximized sensitivity (76.9%) and specificity (90.0%) for identifying the odds of death (AUC = 0.9; Table 3).

### RRT data

Median volume of blood processed and median UF rate were 40 (range: 5.4–134) L and 13 (range: 6.7–35) mL/kg/h respectively. Clotting in the filter was evident in 11/116 (9.5%) and suspected in 48/116 (41.4%) RRT treatments. Filter replacement was necessary in 8 treatments (evident clotting n = 6, suspected clotting n = 2) representing 6.9% (8/116) of all RRT treatments. Additional heparin bolus was administered in all clotting events, representing 59/116 (50.9%) of all RRT treatments. Median heparin dose was 12 (range: 10–15) UI/kg. Heparin data were missing for 2 treatments with suspected clotting in the filter.

### Biochemical blood parameters

**Fig. 2:**
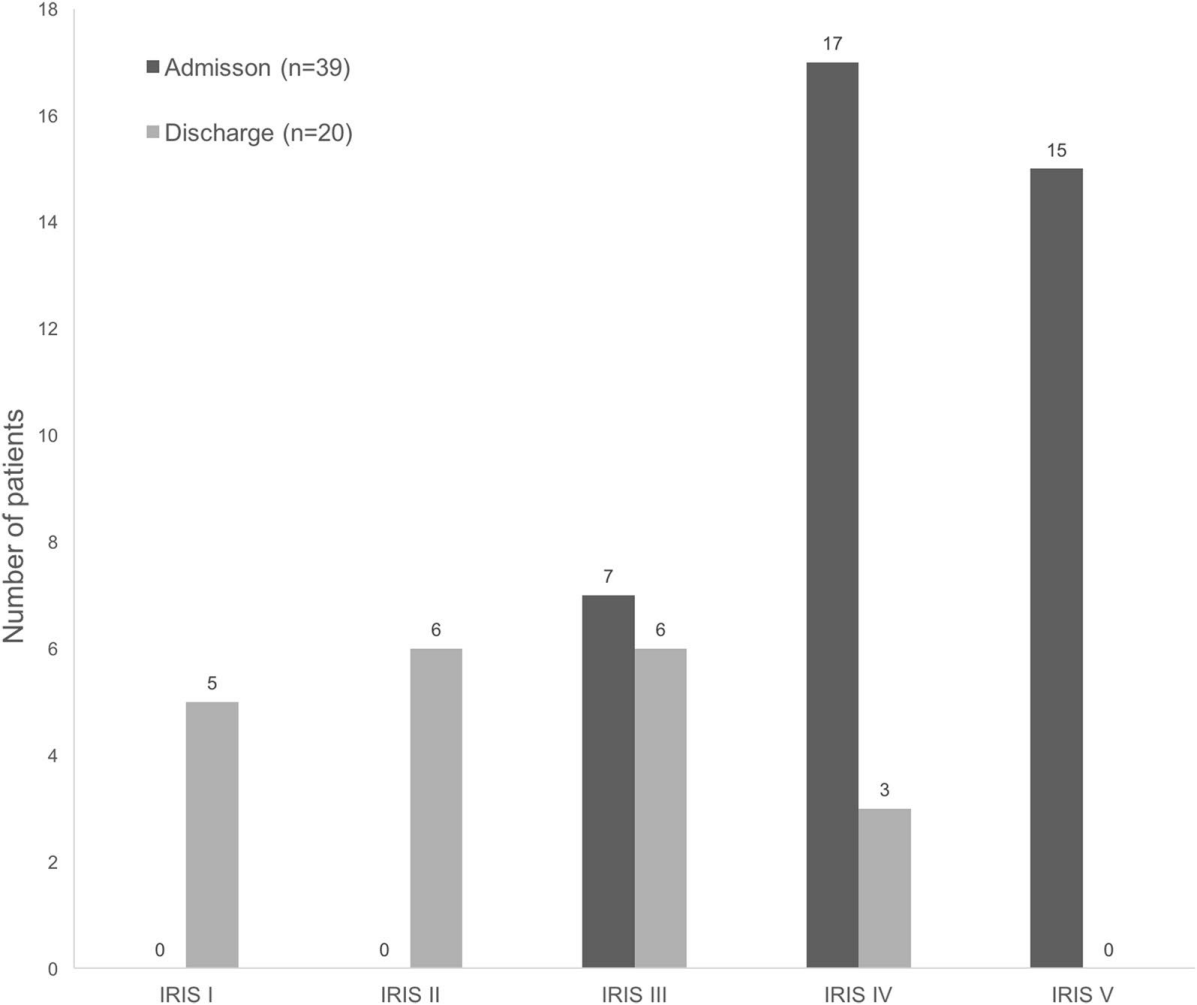
Repartition of AKI grade in 39 dogs with acute kidney injury at admission and at discharge for survivors (20 dogs) using the International Renal Interest Society (see footnote 1) classification. Grade I: creatinine < 140 µmol/L; Grade II: 141 < creatinine < 220 µmol/L; Grade III: 221 < creatinine < 439 µmol/L; Grade IV: 440 < creatinine < 880 µmol/L; Grade V: creatinine > 880 µmol/L

Biochemical blood parameters at admission are summarized in Table 1. Dogs categorized according to the IRIS AKI grading scheme were as follows: Grade V (38%, 15/39), Grade IV (44%, 17/39), and Grade III (18%, 7/39; Fig. 2). URR and CRR were 38.5% (range: 3–55) and 28% (range: 2–97) for the first, 31% (range: 3–65) and 23% (range: 1–97) for the second, 26% (range: 4–61) and 29% (range: 7–79) for the third RRT treatment, respectively. No significant differences were noted in those parameters between the S and NS dogs (P > 0.5 for each). Dogs classified as overfilled had the lowest URR and CRR whereas normovolemic dogs had the highest URR and CRR.

**Fig. 3:**
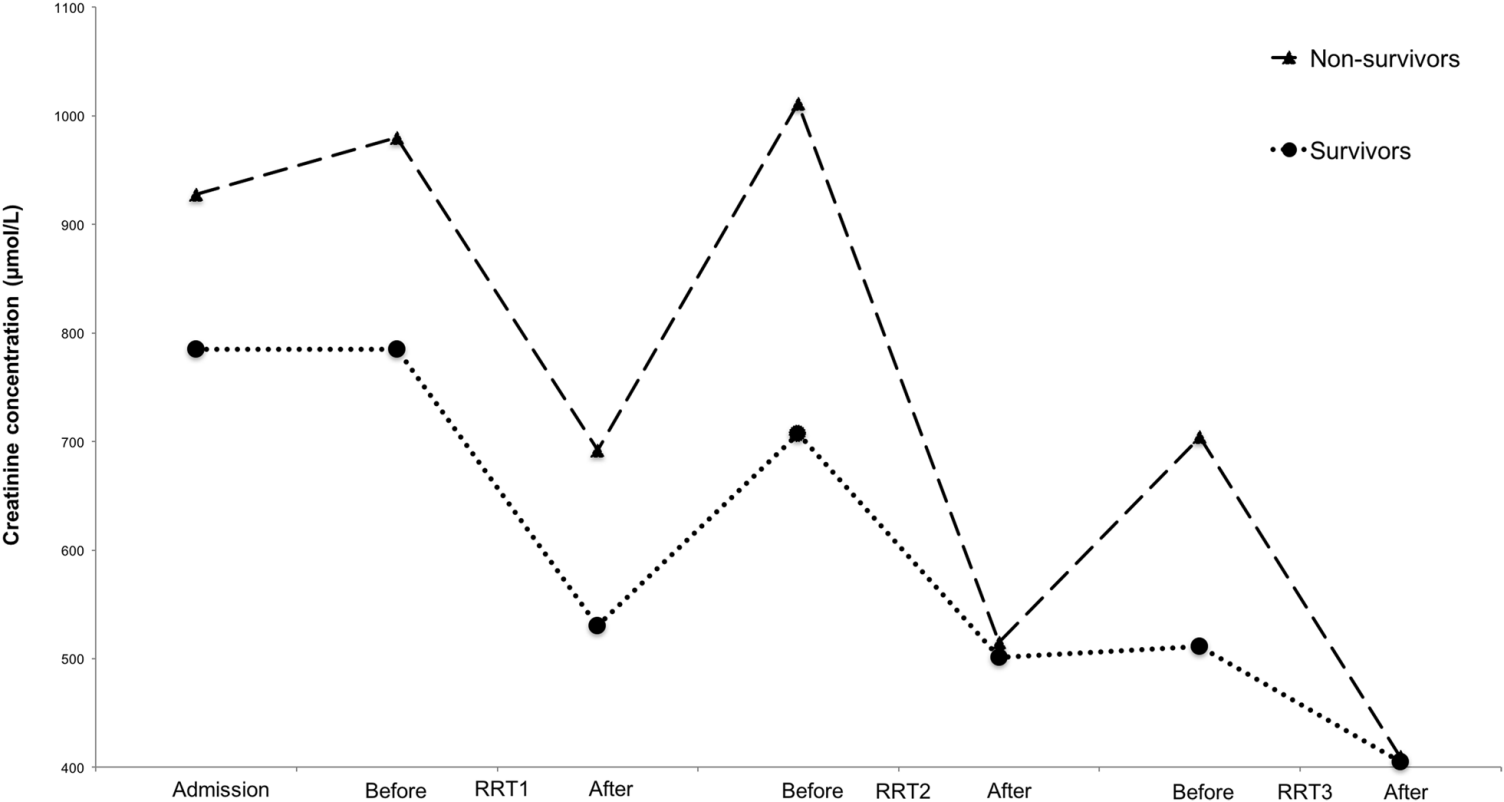
Daily serum creatinine concentration (median) for survivors (dotted line, circle mark) and non-survivors (broken line, triangle mark) during hospitalization. *Significant difference; ^✝^predictive capacity in DeltaCreat/h, defined as the difference between serum creatinine after RRT treatment N and before treatment N + 1 divided by the time (in hours) between the 2 treatments

The median creatinine variation over time is plotted in Fig. 3. During hospitalization, the median DeltaCreat/h on each day was significantly higher in the NS group than in the S group (Table 2). In the multivariate analysis, the DeltaCreat/h (1, 2) between the first and second RRT treatments was associated with a negative outcome (OR = 1.2; 95% CI 1.01–1.35). Based on the ROC curve analysis, the optimal DeltaCreat/h between first and second RRT treatment cut-off value of 1.83 µmol/L/h maximized sensitivity (92%) and specificity (63%) for identifying the odds of death (AUC = 0.8; Table 3).

### Electrolytes and blood gases

In the univariate analysis, the anion gap at admission was significantly higher (29 mmol/L) in NS dogs than in S dogs (25.4 mmol/L, P = 0.01). In the multivariate analysis, only the serum potassium concentration on day 2 was statistically significantly different between groups. Elevated serum potassium concentrations were associated with a negative outcome (OR = 1.9; 95% CI 1.04–3.67). Based on the ROC curve analysis, the optimal serum potassium at day 2 cut-off value of 5.3 mmol/L maximized sensitivity (53%) and specificity (90%) for identifying the odds of death (AUC = 0.7; Table 3).

### Outcome

The overall survival rate was 54% (21/39). Eight dogs (44%) were euthanized (diagnosed with leptospirosis [5/8], leishmaniasis [1/8] or intoxication [2/8]), after a median of 4 (range: 2–7) RRT treatments and 6 (range: 2–6) days.

Among the 10 dogs (56%) experiencing natural death, the causes of death were pulmonary haemorrhages or haemorrhagic diatheses (6/10), cardiac arrhythmia (2/10), pulmonary oedema in one patient with pre-existing heart disease (1/10) and cardiopulmonary arrest during RRT priming in one patient that was moribund (1/10). For dogs that died from pulmonary haemorrhages, 4 (66.7%) died overnight, between 4 and 10 h after end of RRT. For the remaining two (33.3%), timing was not recorded.

Creatinine concentrations at discharge were available for 20/21 survivors. Dogs were categorized according to the IRIS AKI grading scheme (see footnote 1) as follows: grade IV (3/20), Grade III (6/20), Grade II (6/20), Grade I (5/20) (Fig. 2). For the 3 patients with severe azotaemia (IRIS Grade IV), the reasons for hospital discharge were related to financial constraints.

## Discussion

To the authors’ knowledge, this study is the first to describe an intermittent low efficiency haemodiafiltration protocol used in a veterinary ICU. While the question of whether the choice of RRT modality affects patient outcomes has long been a subject of controversy [27], studies have failed to demonstrate a difference in mortality between modalities [28, 29]. Intermittent haemodialysis is a modality that is defined by short, efficient sessions administered at variable intervals, typically for 3–6 h per treatment. Advantages are prompt therapeutic effects and short treatment period, which allows for diagnostic interventions, surgery, and mobilization of patients. The most common complication of IHD is hypotension, which in critically ill, haemodynamically unstable patients, may lead to decompensation, and potential further organ ischaemia and injury [30, 31]. In comparison to IHD, CRRT are intended to run for 24 h per day. Since fluids are removed more slowly, CRRT may result in better haemodynamic stability and better control of fluid balance, which represent an advantage in critically ill patients [32]. Because of its efficient use of fluids, CRRT units use prepackaged fluids, eliminating the need for costly water purification systems that are needed for IHD [5]. Disadvantages of CRRT are the need for immobilization, the use of continuous anticoagulation, and higher costs per treatment. The need for highly specialized 24-h care for CRRT will likely limit the availability of this modality to a very small number of veterinary institutions. As only few centres in Europe and none in France provided RRT in veterinary patient, we decided to establish our program. Because of the high investment necessary for water treatment, associated with precise water quality control protocols and the need of a dedicated space, IHD was not an option in our facility. On the other side, a pure CRRT technique was neither an alternative, considering the personal requirement for the highly specialized 24-h care. In this context, we had to develop a protocol that met our needs, combining advantages of both technique while limiting inconvenient of each. Thanks to the use of pre-packaged sterile fluids, this mobile machine may be moved from cage to cage, without the need of moving unstable patient. Moreover, providing RRT directly in the critical care unit reduce costs, by eliminating the need for a dedicated space, and by sharing vital signs monitor and laboratory analysers as well as veterinary doctors and technicians. Thus, the CRRT platform suited the needs of our veterinary critical care unit, similarly to the ones in human ICU [1]. On the other side, 4 to 5-h treatments appeared to be reasonable, bearing in mind the complexity and costs of care associated with longer duration. By comparison, our protocol of intermittent low efficiency haemodiafiltration appeared to be similar to slow low efficiency diafiltration techniques, which has been shown to provide stable renal replacement therapy in human patients [33]. The shortcomings and disadvantages of our protocol include a lower efficiency in comparison to IHD and costs of filter and pre-packaged fluids similarly to CRRT. In the context of potential coagulopathy, the short duration of RRT treatment in our protocol offered the advantage to limit exposure to anticoagulants. As an integral part of our standardization process, anticoagulation is delivered through a fixed protocol with minimal heparinizing. While different methods to provide anticoagulation during RRT has been published [23, 34], the one described in the present study appeared to be close to heparin-free dialysis protocol previous published [35, 36]. Considering the use of dialysis membranes coated with anti-coagulants, such as heparin-binding to surface-treated AN69, and high probability of coagulopathy in a high number of our dogs, our no to low anti-coagulation protocol tally with actual recommendations [5, 23, 35]. In the present study, clotting in the filter was evident in 9.5%, and suspected in 41.4%. representing almost 50% of all RRT treatments. Mild, moderate and severe clotting in hollow fibre dialyzers has been reported in multicentre study to represent 34%, 17% and 11% of all RRT treatments respectively [23], which is similar to our results. Despite the number of coagulation events, only few (6.9%) were severe enough to require a filter change-out. To decrease clotting events, and at the cost of increased complexity, adaptation to the protocol may be considered, as increasing pre-filter replacement fluid rate (to dilute blood before reaching the filter), the use of continuous heparin administration, or citrate anticoagulation [34].

With regard to treatment delivery, our program seems to be the first in which the full operational responsibility for treatment delivery is assumed by critical care veterinarian and specialized nurses directly in one veterinary ICU. As expected, it has been difficult for some staff to familiarize themselves with conceptually new machinery, although most have been able to attain a degree of proficiency sufficient to manage treatments without hands-on assistance from dedicated haemodialysis personnel. In this context, our 4-h intermittent low efficiency haemodiafiltration protocol appeared to be a valuable option to provide RRT to unstable patients in a veterinary critical care unit, by combining many advantages whilst limiting some drawbacks of both IHD and CRRT.

While demographic data were comparable to those in previous studies [3], our study confirm that BW, sex and breed were not associated with outcome [12, 37]. Although NS dogs were significantly older than S dogs, age was not correlated with outcome in the multivariate analysis, as confirmed by the results of a recent study [38]. The causes of AKI requiring RRT in our canine patient population were similar to those reported in previous studies, with leptospirosis being the most common cause, followed by intoxication [10]. Interestingly, our percentage of cases with leptospirosis of 74% was higher than the 21–30% reported for dogs [12, 14, 39]. This difference may be attributed to regional specificity or referral bias. Even if the rate of intoxications not related to ethylene glycol is similar to previously published data, the antifreeze ban in our country may explain the few ethylene glycol intoxication cases in our population [12, 40]. Whereas other studies documented a correlation between aetiology and survival rate, our study did not confirm this finding [12, 39, 41]. Regarding hospitalization length and the number of RRT treatments being associated with mortality, our results agree with those of one study [3] but disagree with another [37]. Longer hospitalization length was associated with a positive outcome, probably because critically ill or worsening patients are more likely to die or be euthanized earlier. An increased number of RRT treatments was associated with a worse prognosis, with the more severely affected patients probably requiring more RRT treatments in the same hospitalization period; however, this finding remains to be confirmed in further studies, as the prescription of any additional RRT treatment was at the attending clinician’s discretion.

UOP was significantly lower in the NS group at admission and on each day of hospitalization. These results confirmed that oligoanuric AKI is associated with a poor prognosis [12, 42–44]. As suggested by Legrand et al. [45], decreased UOP appeared to be a marker of potential positive fluid balance and risk of fluid overload, which was a determinant in critically ill AKI human patient outcomes. Despite a statistical difference between groups in our study, admission UOP did not predict outcome in the multivariate analyses. However, UOP at day 3 was, in a predictive capacity (AUC = 0.9), comparable to that of published models with AUCs < 0.91 [12] and < 0.8 [4]. While admission parameter may be important initially, hospitalization parameter may be of valuable interest for the clinician, as a negative factor may indicate the need for a more aggressive treatment. On the contrary, if a prognostic indicator was suggestive of a potentially favourable outcome, it may convince disincentive owner to pursue medical care. In our study, several possible explanations may exist for the association of increased UOP at day 3 with higher survival likelihood. First, it has been previously shown in animals that tubular damage was more pronounced in oligoanuric kidneys [46]. Second, it is likely that anuria occurs in the context of multi-organ failure and critical illness, which might be considered in many of our patients (e.g., leptospirosis and leishmaniasis) [43, 47]. Third, maintained UOP may directly benefit the outcome, as it is easier to control volume status and homeostasis [45].

In conventional AKI management, creatinine serum concentration is used to classify severity and is also considered an excellent predictive tool [4, 48]. However, creatinine concentration at admission was not identified as a prognostic factor in our study, which is consistent with others’ findings [12, 39]. Moreover, in the context of RRT managed patient, creatinine will dramatically change because of RRT treatments. Thus, we tried to find a parameter that will represent endogenous change in creatinine concentration despite applications of RRT treatments. We hypothesized that inter-treatment increases in the serum creatinine concentration may have reflected the continuous progress of the disease or the absence of an endogenous creatinine clearance. In our study, creatinine kinetics, as represented by the DeltaCreat/h, was a prognostic factor. As established by the urea kinetic model, variation of timing in post-treatment sampling may cause imprecision, rebound is thought to be largely complete after 30 min, with reported creatinine variation lower than 15% over a 2-h interval [49–51]. Moreover, rebound has been suggested to depend on patient-related mechanisms such as intra-extracellular osmotic fluid shifts, changes in cardiac output or perfusion caused by blood volume changes, cardiac disease or vasoactive drugs [49]. Thus, DeltaCreat/h, as defined in the present study, appeared to a prognostic factor. Strengths of this parameter are the ease of calculation and the high predictive capacity, which was similar to that of multiparametric published models [12, 39]. Without external validation, this parameter should be applied to other populations with caution, and further studies are required.

Electrolyte and blood gas abnormalities are commonly reported in AKI [52]. To date, only serum phosphorus concentration and anion gaps are correlated with mortality in canine AKI [2, 12]. In our study, increased potassium concentration on day 2 was a negative prognostic factor. However, considering the intermediate statistical power (AUC = 0.7), the authors advise considering the 5.3 mmol/L cut-off from the statistical analysis as a therapeutic goal rather than an absolute prognostic factor.

In the present study, the 53% overall survival rate is comparable to the 41% to 56% previously reported in dogs with AKI [12, 15]. Similar to our study, prevalence of haemostatic abnormalities has been reported to be around 23% in leptospirosis-infected dogs [17] and 22.5% in canine AKI [39]. Finally, involvement of two or more systems has been associated with a negative outcome [38, 39, 53], which was the case in the present study for leptospirosis-infected dogs (unpublished data).

Among the survivors, 30% fully recovered with no azotaemia at discharge. In the present study, 55% of survivors were classified as IRIS grade ≤ II at discharge, which is comparable to the previously reported 59% [37]. Because renal recovery to a level compatible with an acceptable quality of life and RRT weaning are essential, our results provide information on expected renal recovery.

Despite our expectations, no significant difference or correlation was demonstrated between referral or RRT initiation time and outcomes. As previously suggested, avoiding or delaying RRT was associated with increased mortality [47, 54–56]. To our knowledge, human studies have failed to establish a correlation between time to RRT initiation and patient outcome, possibly because a reference definition of ‘early’ and ‘late’ initiation is still lacking [57]. Although we used a clinically relevant definition for the time between clinical signs to referral, this may not represent the real time between AKI occurrence and RRT initiation. Even if a potential benefit may exist for early RRT initiation, no clear guidance can be given from our study results, and further studies are needed [58].

Limitations of this study include its retrospective nature and the population size. Despite standard protocols at our facility, treatments may have varied, as they relied on the primary clinician’s discretion. In addition, unassessed parameters may have provided interesting information. Urine-specific gravity, which is still frequently used to evaluate renal function, was not included in our study. While it is still recommended in the veterinary literature, human nephrologists do not emphasize urine-specific gravity when diagnosing AKI, as it solely relies on an increased creatinine concentration or decreased UOP [59, 60]. Fluid overload, another unassessed parameter, may have been of interest, as it was reported as an independent risk factor for mortality in one veterinary study [61].

## Conclusions

This is the first description in the veterinary literature of a 4-h protocol of intermittent low efficiency haemodiafiltration to provide RRT in a veterinary critical care unit. While this protocol appears promising, the clinical application of this protocol requires further investigation. Most dogs were male with leptospirosis-associated AKI or suffering from nephrotoxins other than ethylene glycol. Among parameters associated with survival, UOP was significantly higher in survivors at admission and during hospitalization. Of the other variables, UOP at day 3 and DeltaCreat/h between the first and second RRT treatments may be prognostic indicators. The applicability of these parameters to other populations is unknown, and further international, multicentre prospective studies are warranted to confirm these preliminary observations.

## Abbreviations

AKI: acute kidney injury
BUN: blood urea nitrogen
BW: body weight
CI: confidence intervals
CKD: chronic kidney disease
CVVHDF: continuous venovenous-haemodiafiltration
CRR: creatinine reduction ratio
CRRT: continuous renal replacement therapy
ELISA: enzyme-linked immunosorbent assay
ICU: intensive care unit
IHD: intermittent haemodialysis
IRIS: International Renal Interest Society
NS: non-survivors
OR: odds ratio
PCO2: partial pressure of carbon dioxide
ROC: receiver operating characteristic
RRT: renal replacement therapy
S: survivors
UF: ultrafiltration
UOP: urine output
URR: urea reduction ratio

## Footnotes

1 International Renal Interest Society, IRIS, http://www.iris-kidney.com/pdf/4_ldc-revised-grading-of-acute-kidney-injury.pdf (Accessed October 11th, 2018).

2 Snap Canine Leishmania Antibody Test Kit, IDEXX Laboratories Inc., Westbrook, ME.

3 Urinary catheter, Teleflex Medical, Westmeath, Ireland.

4 Veterinary dialysis catheter, 7.5-French, dual-lumen dialysis catheter, Mila International Inc., Erlanger, KE, or Hemocath, 11.5-French, 24-cm, dual-lumen catheter; Medcomp, Harleysville, PA.

5 Prismaflex System, Gambro Hospal, Meyzieu, France.

6 Hemosol-B0, Gambro Hospal, Meyzieu, France.

7 Prismaflex M60 set (106696; circuit blood volume: 93 mL AN69 HF membrane; surface area: 0.6 m^2^) if BW < 10 kg or M100 set (106697; circuit blood volume: 152 mL AN69 HF membrane; surface area: 0.9 m^2^) if BW > 10 kg, Gambro Hospal, Meyzieu, France.

8 NaCl 0.9%, CEVA Santé Animale, France.

9 Heparine Choay 25,000 UI/mL, Sanofi-Aventis, France.

10 GraphPad Prism version 6, GraphPad Software, Inc., La Jolla, CA.
